# *Plasmodium falciparum* uses vitamin E to avoid oxidative stress

**DOI:** 10.1186/s13071-017-2402-3

**Published:** 2017-10-10

**Authors:** Rodrigo A. C. Sussmann, Wesley L. Fotoran, Emilia A. Kimura, Alejandro M. Katzin

**Affiliations:** Department of Parasitology, Institute of Biomedical Sciences, University of São Paulo, São Paulo, Brazil

**Keywords:** Malaria, *Plasmodium falciparum*, Oxidative stress, Tocopherol, Usnic acid, Antimalarials

## Abstract

**Background:**

*Plasmodium falciparum* is sensitive to oxidative stress in vitro and in vivo, and many drugs such as artemisinin, chloroquine and cercosporin interfere in the parasite’s redox system. To minimize the damage caused by reactive radicals, antioxidant enzymes and their substrates found in parasites and in erythrocytes must be functionally active. It was shown that *P. falciparum* synthesizes vitamin E and that usnic acid acts as an inhibitor of its biosynthesis. Vitamin E is a potent antioxidant that protects polyunsaturated fatty acids from lipid peroxidation, and this activity can be measured by detecting its oxidized product and by evaluating reactive oxygen species (ROS) levels.

**Results:**

Here, we demonstrated that ROS levels increased in *P. falciparum* when vitamin E biosynthesis was inhibited by usnic acid treatment and decreased to basal levels if exogenous vitamin E was added. Furthermore, we used metabolic labelling to demonstrate that vitamin E biosynthesized by the parasite acts as an antioxidant since we could detect its radiolabeled oxidized product. The treatment with chloroquine or cercosporin of the parasites increased the ratio between α-tocopherolquinone and α-tocopherol.

**Conclusions:**

Our findings demonstrate that vitamin E produced endogenously by *P. falciparum* is active as an antioxidant, probably protecting the parasite from the radicals generated by drugs.

**Electronic supplementary material:**

The online version of this article (10.1186/s13071-017-2402-3) contains supplementary material, which is available to authorized users.

## Background

Malaria remains one of the most important infectious diseases globally. In 2015, 214 million cases of malaria and 438,000 deaths were reported [1]. One of the problems of malaria control is the emergence and spread of *P. falciparum* strains resistant to available antimalarials. Artemisinin derivatives were the only drug that did not present a disseminated resistance, but in 2014, a molecular marker for artemisinin resistance was identified [2]. In view of this problem, several groups have been working to identify new targets for antimalarials.

The apicoplast, an organelle present in most apicomplexans, including *Plasmodium* spp.*,* is a non-photosynthetic plastid homologous to the chloroplasts of plants and algae that harbors pathways with similarities to those in plants plastids and cyanobacteria, including the 2-C-methyl-D-erythritol 4-phosphate (MEP) isoprenoid biosynthesis pathway [3, 4]. In 2011, Yeh & DeRisi [5] demonstrated that the only essential apicoplast function is to supply the demand of isopentenyl pyrophosphate (IPP) in *P. falciparum*. Because the isoprenoids are biosynthesized in humans by the mevalonate pathway, the MEP pathway is an interesting target against which to develop new antimalarials. Our group previously demonstrated that MEP pathway is active in the intraerythrocytic stages of *P. falciparum* [3] and synthesizes dolichol of 11–12 isoprenic units [6], ubiquinone [7], dolichylated/ isoprenylated proteins [8, 9], carotenoids [10], vitamin E (α-tocopherol) [11] and menaquinone [12].

*Plasmodium falciparum* is sensitive to oxidative stress in vitro and in vivo, and a number of drugs act on the parasite redox system. Examples of these drugs are artemisinin [13], chloroquine [14] and cercosporin [15]. To minimize the damage caused by reactive oxygen (ROS) or nitrogen (RNS) species, enzymes of the antioxidant pathway must be functionally active [16].

Several enzymes of the glutathione system have been described in *Plasmodium* species. These include glutathione synthase [17] and reductase [18], superoxide dismutase [19], glutamate dehydrogenase [20] and glucose 6-phosphate dehydrogenase [21]. Additionally, the parasite has a functional thioredoxin system with thioredoxin reductase [22], thioredoxin [23], thioredoxin peroxidase [24] and 1 *cys*-peroxyredoxin [25] and α-tocopherol [11].

Final products of the isoprenoid biosynthesis such α-tocopherol may act as antioxidants and in membrane stabilization, as described in plants [26] and cyanobacteria [27]. However, to support the hypothesis that the role of α-tocopherol biosynthesized by the parasite is an active antioxidant in the redox systems, it is necessary to detect its oxidized product (α-tocopherolquinone) and evaluate ROS levels.

α-Tocopherol was shown to be synthesized by all replicative intraerythrocytic stages via the isoprenoid pathway [11]. This biosynthesis of α-tocopherol is inhibited by usnic acid and increases if the parasite is cultivated in a higher oxygen tension [11]. Accordingly, the lipid peroxidation increases in *P. falciparum* culture treated with usnic acid [11]. This is expected since the main function of vitamin E is to avoid autoxidation of polyunsaturated fatty acids [28, 29].

Given the fact all the previous observations indicated that vitamin E is involved in redox protection of membranes, we set out to directly prove the presence of oxidized intermediate of this molecule, further underlining its antioxidant role in *Plasmodium* blood stage metabolism.

## Methods

### *Plasmodium falciparum* culture

The *P. falciparum* strain 3D7 was cultured in vitro according to Trager & Jensen [30] with modifications [11]. The cultures (approximately 15% parasitemia) were initially synchronized in ring stages (6–22 h after the invasion) by treatment with 5% (*w*/*v*) D-sorbitol solution in water. The parasites were maintained in culture until the development of trophozoite (26–34 h after reinvasion) or schizont (38–48 h after reinvasion) stages and then synchronized by gelatin flotation [31]. Parasite development was monitored by daily microscopic evaluation of Giemsa-stained thin smears.

### Fluorescence microscopy

Uninfected and infected erythrocytes cells with *P. falciparum* were stained with 5 μM CellRox for 30 min in PBS. In the last 5 min of incubation, DAPI nucleic acid stain was added to a final concentration of 200 nM. As a positive control, the same procedure was done in parallel with uninfected and infected erythrocytes incubated with 0.5 μM H_2_O_2_. The cells were washed 3× with phosphate-buffered saline (PBS, 30 mM Na_2_HPO_4_, 6 mM KH_2_PO_4_, 120 mM NaCl, pH 7.4), mounted on glass slides and coverslips and fluorescence microscopy images were acquired on a camera (Axio Cam HRc, Zeiss, Göttingen, Germany) connected to an optical microscope (up to 100×) (Axio Imager M2, Zeiss, Göttingen, Germany). Fluorescent filters used were 02 DAPI (358 nm/463 nm) and 63 HE MRFP (585 nm/608 nm) (Excitation/Emission, respectively).

### ROS levels

The cell-permeant CellRox deep red (Molecular Probes®, Eugene, USA) reagent is sensitive to superoxide anion ($\mathrm{O}{\cdot}_2^{-}$) and hydroxyl radical (^•^OH) and was used to measure the ROS levels. Uninfected and infected erythrocytes with *P. falciparum* were stained with 5 μM CellRox for 30 min in PBS. In the last 5 min of incubation, SYTO 16 *Green* Fluorescent Nucleic Acid Stain (Molecular Probes®) was added to a final concentration of 40 nM. The same procedure was run in parallel with uninfected and infected erythrocytes incubated with 0.5 μM H_2_O_2_ (positive control) or 0.5 μM H_2_O_2_ plus 75 μM α-tocopherol (negative control) 30 min before the CellRox addition. The cells were washed 3× with PBS and analyzed by flow cytometry in a Guava Easycyte Mini System (Millipore, Billerica, USA) for the fluorescence measured with excitation wave length 488 nm and emission filters of 680/40 nm (red) and 525/30 nm (green). The levels of ROS were measured in untreated and treated cultures with the half-maximal inhibitory concentration (IC_50_) of usnic acid (25 μM) for 48 h before the analysis [11].

### Oxidation of α-tocopherol standard

The oxidation of α-tocopherol was performed as described by Liebler et al. [32]. α-tocopherolquinone was obtained from the oxidation of 4 mg α-tocopherol by addition 0.15 mM Cumene hydroperoxide (CumOOH) and 0.1 mM ferrous ammonium sulphate (Fe(NH_4_)_2_(SO_4_)_2_) in a final volume 200 μl 50 mM Tris, pH 7. After 30 min incubation at 37 °C, the oxidation was stopped by adding up to 1 mM deferoxamine mesylate, and the mixture was extracted three times with 1 ml hexane. The hexane extract was evaporated and submitted to RP-HPLC and GC-MS analysis.

### Reversed phase-high-performance liquid chromatography (RP-HPLC)

The stationary phase was a Phenomenex Luna C18 column (250 mm × 4.6 mm × 5 μm) (Phenomenex, Torrance, USA) coupled to a pre-C18 column (Phenomenex, Torrance, USA), a diode array detector (DAD) type Gilson 170 (270 and 295 nm) and a fraction collector FC203B. The software used for data processing was the Trilution™ LC 3.0 System Software. An isocratic system with 1 ml/min methanol was used, and the fractions were collected per minute [32]. The resulting fractions were dried, resuspended in 600 μl of liquid of scintillation mixture (Perkin-Elmer Life Sciences, Waltham, USA) and the radioactivity were monitored with a Beckman 5000 β-radiation scintillation counter (Beckman, Los Angeles, USA).

### Gas chromatography - mass spectrometry (GC-MS)

A Y2K ion trap mass spectrometer (MS) PolarisQ System (Finnigan ThermoQuest Inc., San Jose, USA) equipped with a nano-source type electron impact ionization (EI) was used. A TRACE GC equipped with a 30 m × 25 mm × 0.25 μm DB-5 ms column and 1 ml/min helium as mobile phase was used.

The analysis conditions were an initial oven temperature of 120 °C for 4 min then a ramp of 20 °C/min until 300 °C. This temperature was maintained for additional 3 min. The injector and transferline temperatures were 215 °C and 275 °C, respectively. The ion source was maintained at 200 °C, and 2 segments were acquired by first monitoring a range of *m/z* 50 to 500 (fullscan) and then a second monitoring of the fragmentation (MS/MS) of the ion at *m/z* 446. The excitation energy scale for fragmentation was 0.225 and 0.85 V. The monitored fragment ions of tocopherolquinone were at *m/z* 150, 203, 221 and 428 [32]. The mass spectra were analyzed using the Xcalibur data analysis program, version 1.3.

### Inhibition growth assay in vitro of *P. falciparum*

The method described by Desjardins et al. [33] was employed to determine the IC_50_ in 48 h of cercosporin (Sigma-Aldrich, St. Louis, MO) in *P. falciparum* cultures. Cercosporin was diluted in dimethyl sulfoxide (DMSO) and then serially diluted in culture medium to reach the desired concentrations. Two controls were used, one with no treatment and another with the solvent in which the drug had been diluted. These tests were performed in cell culture 96-well plates (Eppendorf, Hamburg, Germany). Daily thin blood smears were made to control parasitaemia.

### Metabolic labelling

Synchronous cultures of *P. falciparum* in trophozoite stage were metabolically labeled with 0.75 μCi/ml of [1(n)- ^3^H]-geranylgeranyl pyrophosphate ([^3^H]GGPP) (14 Ci/mmol, Amersham, Picataway, USA). After 12–16 h, schizont stages were concentrated by the magnetic fractionation process [34]. The volume of the infected erythrocytes was measured, lyophilized and stored in liquid nitrogen for later analysis. In parallel, cultures were treated for 48 h with the IC_50_ of cercosporin or chloroquine. In this case, the metabolic labeling occurred between the last 12–16 h of treatment and the schizonts were concentrated as described above. It is important to emphasize the parasite culture was immediately lyophilized after magnetic column concentration and stored in liquid nitrogen to avoid unspecific oxidation of α-tocopherol.

### Extraction of vitamin E and its oxidation products from *P. falciparum*

1.5 × 10^9^ lyophilized erythrocytes infected with *P. falciparum* (300 μl) metabolically labeled as described above were resuspended in 1 ml deionized water in glass tubes. Cell lysis was achieved by ultrasonication in a Branson sonifier with three pulses of 5 s with 10% of potency and 10 s intervals between them at 4 °C. The proteins were precipitated by adding 200 μl ethanol-0.01% butylated hydroxytoluene (BHT). After mixing for 1 min, extraction was done three times with 2 ml of hexane - 0.01% BHT. The sample was mixed for 1 min and centrifuged at 2700*× g* for 10 min at 4 °C [35]. The hexane extract of erythrocytes infected with *P. falciparum* was evaporated and submitted to RP-HPLC analysis. All the procedure was done avoiding light and heat.

### Statistical analysis

Statistical significance was determined by Student’s t-test, one-way ANOVA or nonlinear regression (dose response) analysis using Prism 5.3 software (GraphPad, La Jolla, USA) or Origin 8 (OriginLab Corporation, Northampton, USA).

## Results

### CellRox effectively detected oxidative stress

To determine whether CellRox could effectively visualize oxidative stress levels in *P. falciparum*, fluorescence microscopy was performed. For this, the response of the culture exposed to an oxidative stress source was tested (Additional file 1: Figure S1).

The culture that was not treated with H_2_O_2_ showed a basal level of oxidative stress in infected and non-infected erythrocytes, while the treated ones showed a strong increase in mean fluorescence exclusively in the three stages of the parasite.

### CellRox stained erythrocytes can also be measured by cytometry

**Fig. 1:**
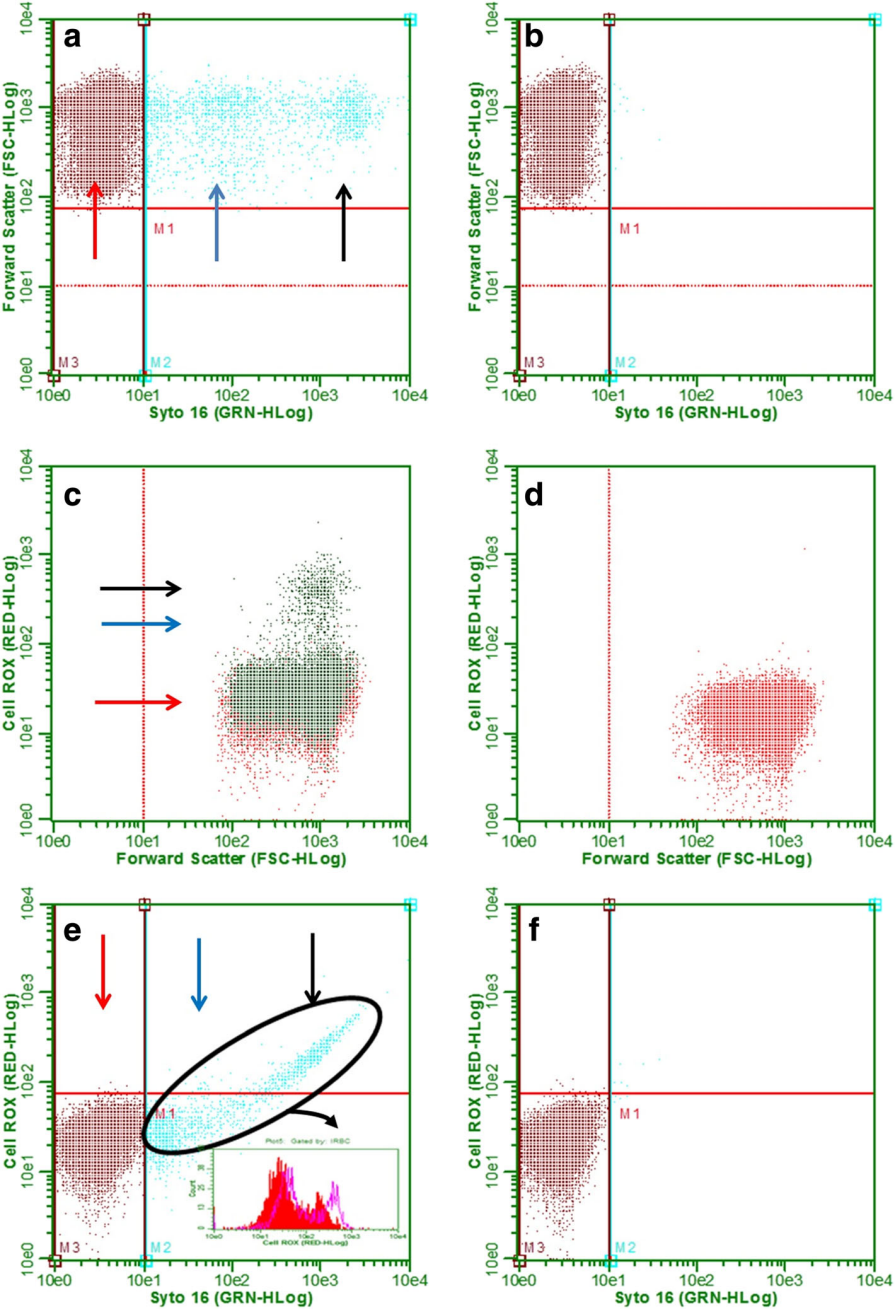
Parameters used in the analysis of ROS levels. **a** Infected erythrocytes stained with SYTO 16 green. **b** Uninfected erythrocytes stained with SYTO 16 green. **c** Infected erythrocytes stained with CellRox. **d** Uninfected erythrocytes stained with CellRox. **e** Infected erythrocytes cells stained with SYTO 16 green and CellRox-histogram comparing the ROS levels in infected erythrocytes challenge (*pink line*) and unchallenged (*red solid*) with H_2_O_2_ in 20 min of incubation with CellRox. **f** Uninfected erythrocytes stained with SYTO 16 green and CellRox. *Red arrow*: uninfected erythrocytes; *blue arrow*: trophozoites; *black arrow*: schizonts; *black circle*: infected erythrocytes population

The two populations observed in parasite cultures stained with SYTO 16 Green were classified as infected and non-infected erythrocytes (Fig. 1a, b). In parasite cultures stained with CellRox, it was observed that erythrocytes with increased granularity (corresponding to infected erythrocytes) showed a highest basal level of oxidation compared with non-infected erythrocytes (Fig. 1c, d). The incubation of infected and non-infected erythrocytes with both probes (Fig. 1e, f) indicated that only in infected erythrocytes is it possible to observe a population with a diagonal displacement (Fig. 1e, black circle). The basal level of oxidative stress in schizonts (Fig. 1e, black arrow) were higher than in trophozoites (Fig. 1e, blue arrow), a fact already described by Butzloff et al. [15]. With these parameters standardized, we analyzed only the infected erythrocytes population in cultures previously challenged with H_2_O_2_.

To determine the time to detecting the difference between the redox state of challenged and unchallenged cultures, the ROS generation was monitored at different times of incubation (Additional file 1: Figure S2). It is possible to observe that the ROS levels in the culture challenge with H_2_O_2_ were higher compared with control in all time course (t-test: *t*_(4)_ = 16.40, *P* < 0.0001). The 20 min of incubation was chosen because at 0 and 10 the ROS levels were increasing and at 30 and 40 min a plateau of ROS levels was reached and presented a higher variation. The histogram in the bottom of Fig. 1e demonstrates that the infected erythrocytes pre-incubated with H_2_O_2_ (pink line) showed a higher level of oxidative stress than the unchallenged infected erythrocytes (red solid) after 20 min of incubation with CellRox.

### Usnic acid treatment increases ROS levels in *P. falciparum*

**Fig. 2:**
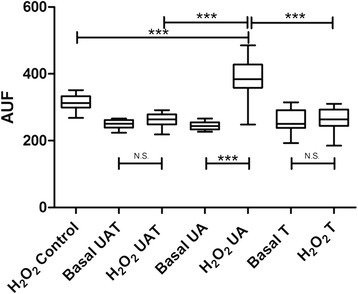
Levels of ROS in erythrocytes infected with *P. falciparum*. Cytometry analysis of infected erythrocytes challenged or not (basal) with the pro-oxidant H_2_O_2_. The levels of oxidative stress were evaluated in different treatments: H_2_O_2_, hydrogen peroxide; UAT, usnic acid plus 75 μM α-tocopherol; UA, usnic acid and T, 75 μM α-tocopherol. *Abbreviations*: AUF: arbitrary unit of fluorescence; N.S.: not significant (ANOVA: *F*_(6,77)_ = 30.6, *P* < 0.0001)

Using the benchmark parameters described above, we analyzed parasite cultures treated in vitro with usnic acid, the inhibitor of α-tocopherol biosynthesis in *P. falciparum* [11] (Fig. 2).

The oxidative stress levels showed no significant difference in the treatments that were not challenged with H_2_O_2_. When parasites were challenged with the pro-oxidant H_2_O_2_, we detected an increase of 63% in oxidative stress levels in the culture treated with usnic acid (H_2_O_2_ UA) when compared with the treated and not challenged culture (Basal UA) (ANOVA: *F*_(6,77)_ = 30.6, *P* < 0.0001). The oxidative stress was maintained in basal levels when 75 μM of vitamin E was added to cultures treated with usnic acid and challenged with H_2_O_2_ (H_2_O_2_ UAT) compared to cultures where no α-tocopherol was added (H_2_O_2_ UA) (ANOVA: *F*_(6,77)_ = 30.6, *P* < 0.0001). (Fig. 2).

### Changes in the ratio of α-tocopherolquinone and α-tocopherol occur as a result of a redox imbalance

Until now, vitamin E biosynthesized by *P. falciparum* was only indirectly implied as an antioxidant [11]. If vitamin E actively participates in the redox system of the parasite, oxidized forms of vitamin E, such as α-tocopherolquinone, should be detectable.

To determine the retention time of α-tocopherolquinone in the RP-HPLC analysis of metabolically labelled parasites, the α-tocopherol standard was oxidized, and its quinone was purified by RP-HPLC (Additional file 1: Figure S3). The fractions resulting from chromatography purification were analyzed by GC-MS to confirm the oxidation of α-tocopherol standard to α-tocopherolquinone. Fraction 29 was identified as α-tocopherol, and fraction 21 had an identical MS/MS spectrum compared to the α-tocopherolquinone present in the GC-MS library (Additional file 1: Figure S4).

The ion at *m/z* 446 corresponds to the molecular mass of α-tocopherolquinone in the GC-MS library (Additional file 1: Figure S4b) is present in the analyzed HPLC fraction 21 (Additional file 1: Figure S4a) and also an ion at *m/z* 428, corresponding to the molecular mass without water. The ion fragmentation profile of ion at *m/z* 446 was identical to the pattern, thereby confirming the identity of the compound eluted in the fraction 21.

**Fig. 3:**
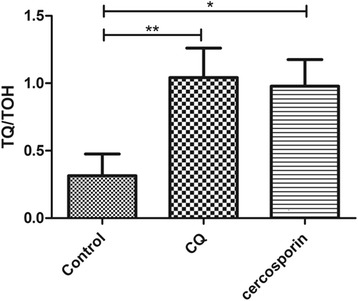
Changes in the ratio of α-tocopherol and α-tocopherolquinone in infected erythrocytes. Ratio between TQ (α-tocopherolquinone) and TOH (α-tocopherol) in control, CQ (chloroquine) and cercosporin (ANOVA: *F*_(2,6)_ = 12.92, *P* = 0.006)

To establish if exogenous sources of oxidative stress (e.g. cercosporin and chloroquine) lead to an imbalance of α-tocopherolquinone and tocopherol ratio, we used chloroquine (IC_50_ 7 nM) and cercosporin (IC_50_ 177 nM). The IC_50_ for cercosporin was calculated for the 3D7 strain (Additional file 1: Figure S5). Once confirmed the elution time of α-tocopherolquinone and determined the IC_50_ of cercosporin, treated and untreated radiolabeled parasite extracts were analyzed by RP-HPLC. The ratio between the radioactivity in the fractions corresponding to the α-tocopherolquinone and α-tocopherol was calculated in infected erythrocytes treated with cercosporin or chloroquine and compared with untreated control cultures (Fig. 3).

The ratio between α-tocopherolquinone and α-tocopherol increased significantly from 0.31 ± 0.16 in control to 0.97 ± 0.19 and 1.04 ± 0.21 in chloroquine and cercosporin treatments, respectively (ANOVA: *F*_(2,6)_ = 12.92, *P* = 0.006). This indicates that an exogenous source of oxidative stress interferes in the pool of α-tocopherol and its oxidized form, probably as a means of maintaining the redox balance.

## Discussion

The genus *Plasmodium* comprises a group of unicellular parasites that have to deal with intense oxidative stress caused by several factors, such as metabolism of haemoglobin,and blast of oxidative radicals characteristic to neutrophils in inflammatory process [36]. Erythrocytic stages of *Plasmodium* are exposed to a number of events that induce oxidative stress [37] and the parasite has developed a number of mechanisms to deal with radicals [38]. Similar to other species, *Plasmodium* disposes of vitamin E to regulate redox events. Our data show that vitamin E biosynthesized by *P. falciparum* via the isoprenoid pathway plays a role in the redox system of the infected erythrocyte. It was shown that the treatment with usnic acid inhibits vitamin E biosynthesis in *P. falciparum* [11] and in our hands this treatment increases the susceptibility to ROS formation when the culture was challenged with the oxidant, leading to oxidative stress (Fig. 2). Similar to this result, in spinach thylakoids, α-tocopherol in combination with vitamin K1 and plastoquinone act as scavengers of superoxide formed by photosystem I in plant photosynthesis [39]. The formation of hydrogen peroxide is inhibited which results in the protection of thylakoid membranes against lipid peroxidation [39].

The presence of the oxidized form of α-tocopherol, α-tocopherolquinone, in *P. falciparum* was characterized by metabolic labelling and RP-HPLC analysis. Chloroquine exerts its effects by hampering hemozoin formation [14], and cercosporin is present in the fungal plant pathogen *Cercospora nicotinae*, and is a natural singlet oxygen generator (^1^O_2_) [40, 41]. Cercosporin has been previously used to measure ^1^O_2_ formation in *P. falciparum* [15]. However, the authors of the study did not describe the IC_50_ of cercosporin for the parasite. Here, we calculated the IC_50_ of cercosporin for *P. falciparum* 3D7 strain.

The ratio between α-tocopherolquinone and α-tocopherol increased in chloroquine or cercosporin treatments (Fig. 3). The same effect was described in plants under water stress [42], high light stress [43] and nutrient deprivation [44]. In animals, this phenomenon is also observed in different types of oxidative stress such as hyperoxia [45], patients who had undergone an aortic cross-clamping operation [46], fatigue induced by overnight desk work [47] and cognitive impairment in elderly people [48].

The redox imbalance and change in α-tocopherol and α-tocopherolquinone ratio reinforces the view that vitamin E biosynthesized by the parasite participates in the redox system present in the infected erythrocyte. However, the treatment with the IC_50_ of usnic acid did not change the IC_50_ values of chloroquine (data not shown). The parasite may possess other mechanisms to deal with redox imbalance when the vitamin E biosynthesis is inhibited. For example, infected erythrocytes lose the ability to biosynthesizes glutathione de novo because the intermediate *γ*-glutamyl-cysteine is depleted from the host cell [49]. This disadvantage is compensated for by the exportation of glutathione oxidized by the parasite into the cytoplasm of erythrocyte, where it is reduced by the glutathione reductase from the cell and by the enzymes of pentose’s cycle [49].

Given the importance of the antioxidant function represented by α-tocopherol and α-tocopherolquinone for the parasite, we investigated if this system could be useful to avoid the oxidative environment. Here, the system was capable of protecting the parasite from elevated oxidative stress (Fig. 2).

The use of α-tocopherol and α-tocopherolquinone as a membrane antioxidant can also be an alternative route to avoid intense oxidative stress caused by immune interactions on membranes of erythrocytes. The hostile environment can change parasite metabolism and permit adaptation in the cell cycle to the parasite adapted to achieve the new invertebrate host in a gametocyte formation [50, 51]. The time to resist hostile environments until infection of the invertebrate host is essential for life-cycle perpetuation, and α-tocopherol and α-tocopherolquinone can be an additional antioxidant system in this process. These hypotheses must be studied.

We showed that α-tocopherol biosynthesized by *P falciparum* is found in its oxidized form as a defence mechanism against oxidative stress, but we cannot discard the idea that α-tocopherol from the host can be used for the same purpose.

## Conclusions

α-tocopherol appears to play an important function as an antioxidant against environmental stress, including maintaining ROS levels. Furthermore, the metabolic pathway of vitamin E biosynthesis could be an interesting target for the development of drugs against *P. falciparum*.

## Additional file

Additional file 1: Figure S1. Imaging evaluation of CellRox. **Figure S2.** Time course of ROS generation. **Figure S3.** RP-HPLC purification of α-tocopherol and its oxidation product. **Figure S4.** GC/MS identification of tocopherolquinone. **Figure S5.** Determination in vitro of inhibitory concentration of 50% of the growth of cercosporin in *P. falciparum. (DOCX 847 kb)*

## Abbreviations

$$ \mathrm{O}{\cdot}_2^{-} $$: Superoxide anion
[^3^H]GGPP: [1(n)- ^3^H]-geranylgeranyl pyrophosphate
BHT: Butylated hydroxytoluene
CumOOH: Cumene hydroperoxide
DAD: Diode array detector
DMSO: Dimethylsulfoxide
FP: Ferriprotoporphyrin IX
GC-MS: Gas chromatography-mass spectrometry
IC_50_: Half-maximal inhibitory concentration
IPP: Isopentenyl pyrophosphate
MEP: 2-C-methyl-D-erythritol 4-phosphate
OH: Hydroxyl radical
PBS: Phosphate-buffered saline
RNS: Reactive nitrogen species
ROS: Reactive oxygen species
RP-HPLC: Reversed-phase high-performance liquid chromatography
