# Supplementary material for: Plasmodium falciparum uses vitamin E to avoid oxidative stress
Source: Parasit Vectors. 2017 Oct 10;10:461. doi: 10.1186/s13071-017-2402-3 (PMC5634829; doi:10.1186/s13071-017-2402-3)
Supplement: Additional file 1: Figure S1. — Imaging evaluation of CellRox. Figure S2. Time course of ROS generation. Figure S3. RP-HPLC purification of α-tocopherol and its oxidation product. Figure S4. GC/MS identification of tocopherolquinone. Figure S5. Determination in vitro of inhibitory concentration of 50% of the growth of cercosporin in P. falciparum. (DOCX 847 kb) [file 13071_2017_2402_MOESM1_ESM.docx]

**Additional file 1**

***Plasmodium falciparum* uses vitamin E to avoid oxidative stress**

Rodrigo A. C. Sussmann, Wesley L. Fotoran, Emilia A. Kimura and Alejandro M. Katzin*

Department of Parasitology, Institute of Biomedical Sciences, University of São Paulo, São Paulo, Brazil.

*Corresponding author: Department of Parasitology, Institute of Biomedical Sciences, University of São Paulo, Av. Lineu Prestes 1374, CEP 05508-000, São Paulo, SP, Brazil. Phone: 55-11-30917330, fax: 55-11-30917417, Email [amkatzin@icb.usp.br](mailto:amkatzin@icb.usp.br)

**
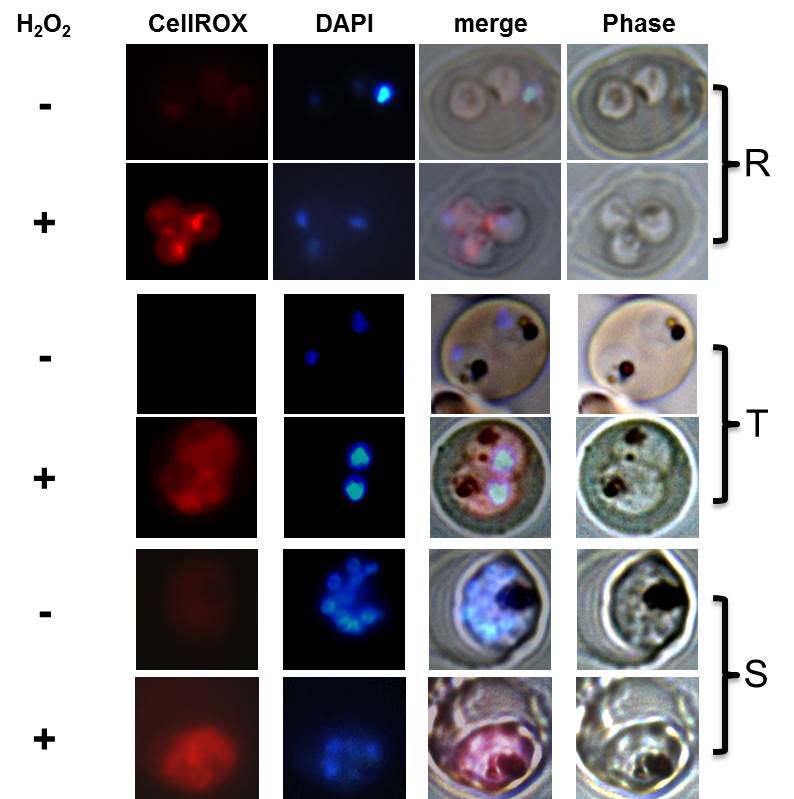
**

**Figure S1. Imaging evaluation of CellRox.** Panel demonstrating that CellRox detects ROS formation in the three stages of *P. falciparum*. The fluorophore has no fluorescence when reduced. When oxidized, it emits a red fluorescence. First column, fluorescence of CellRox stain; second column, fluorescence of DAPI; third column merge and fourth column, Phase. Symbols: (-) absence; (+) presence of H_2_O_2_. Parasite stages: R, ring; T, trophozoite; S, schizont. Original magnification for all images: 1.000×.

**
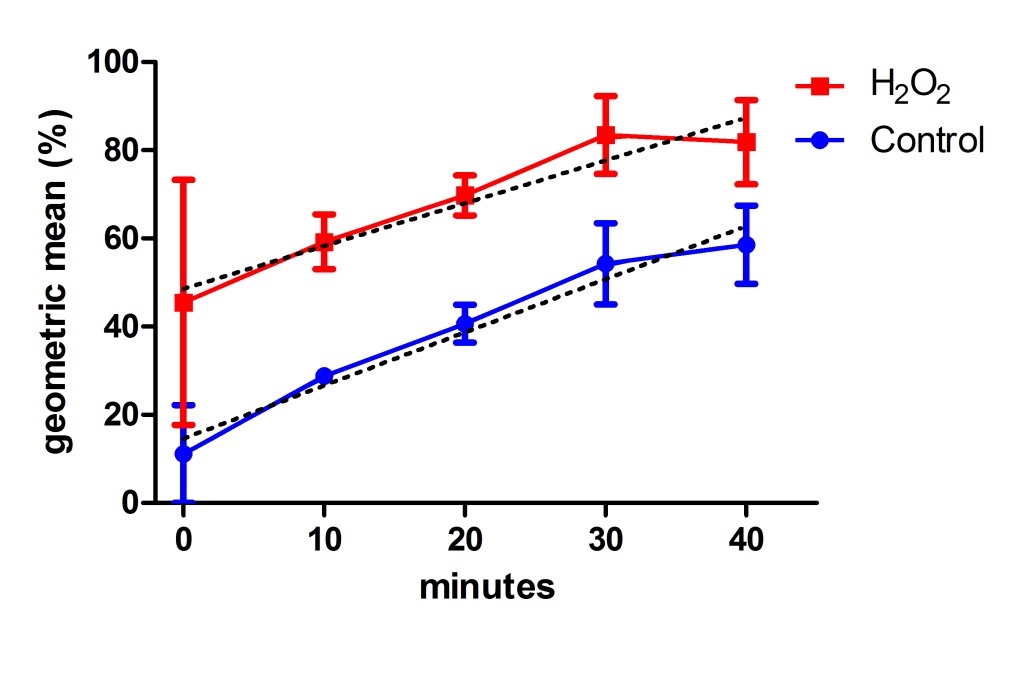
**

**Figure S2. Time course of ROS generation.** Analysis of infected erythrocytes stained with CellRox and Syto 16 green in different times of incubation. The ROS levels were monitored in 0, 10, 20, 30 and 40 min. Red line, culture challenge with H_2_O_2_; blue line, control (t-test: *t*_(4)_ = 16.40, *P* < 0.0001).

**
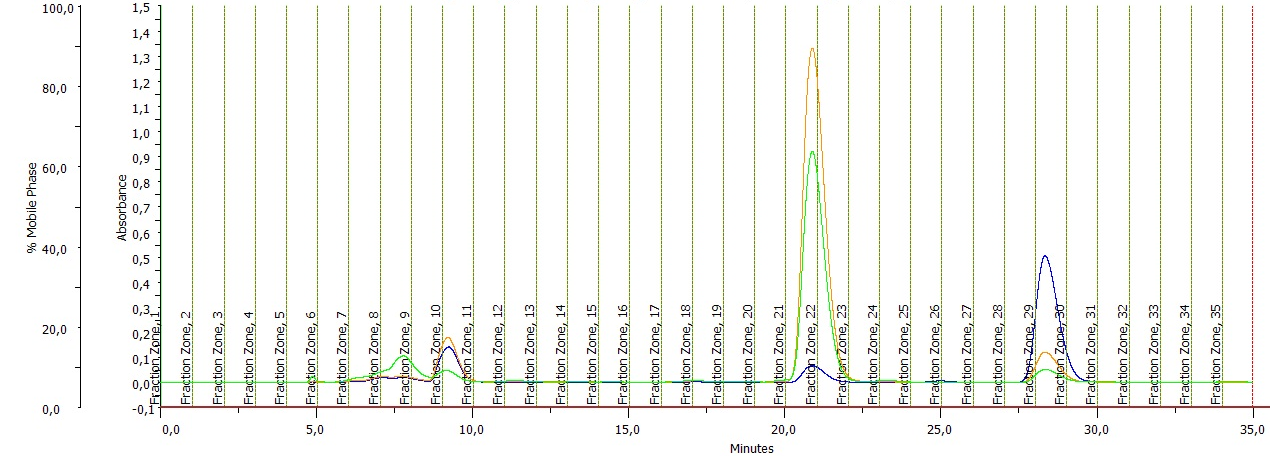
**

**Figure S3. RP-HPLC purification of α-tocopherol and its oxidation product.** The oxidation reaction of α-tocopherol was purified by RP-HPLC. The fraction 29 is the authentic α-tocopherol standard and the fraction 21 is the α-tocopherolquinone. Wavelengths: blue, 210 nm; yellow, 270 nm and green, 295 nm.

**
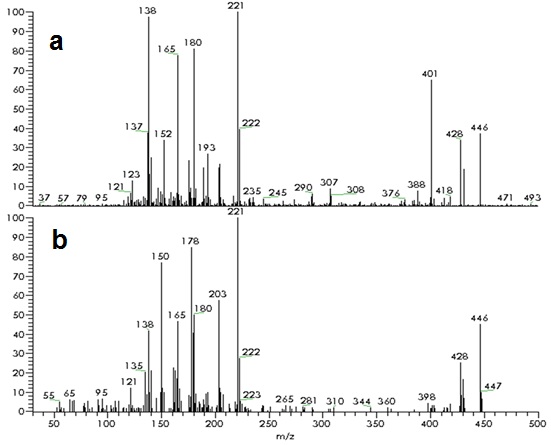
**

**Figure S4. GC/MS identification of tocopherolquinone.** MS/MS spectrum of oxidized α-tocopherol. (a) mass spectrum obtained of RP-HPLC fraction. (b) α-tocopherolquinone mass spectrum present in the software library.

**
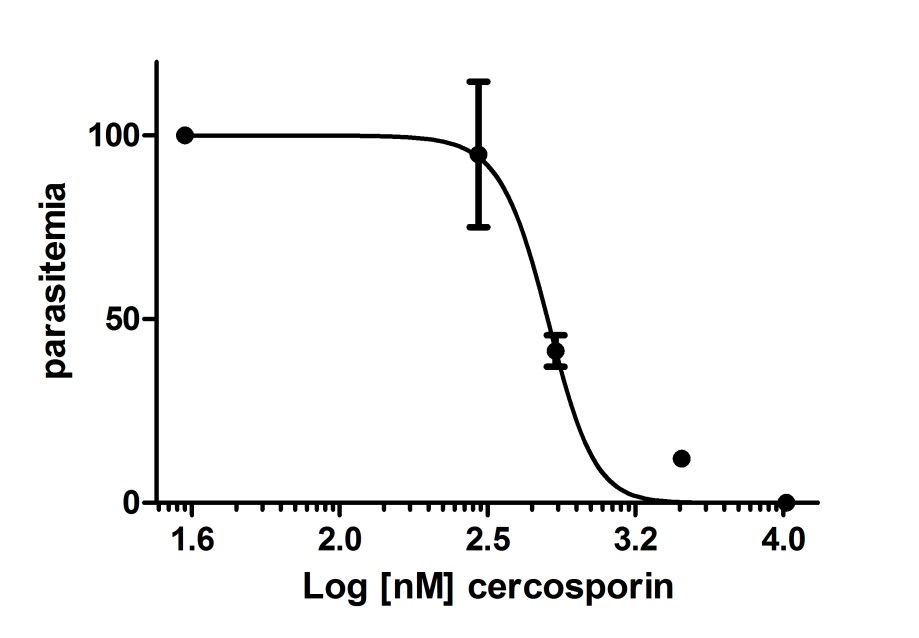
**

**Figure S5. Determination *in vitro* of inhibitory concentration of 50% of growth of cercosporin in *P. falciparum.*** Nonlinear regression dose-response type in 48 h of treatment with different concentrations of cercosporin. IC_50_ = 177±44nM (n=3).
